# Presence and potential distribution of malaria-infected New World primates of Costa Rica

**DOI:** 10.1186/s12936-021-04036-y

**Published:** 2022-01-08

**Authors:** Andrea Chaves, Gaby Dolz, Carlos N. Ibarra-Cerdeña, Genuar Núñez, Edgar Ortiz-Malavasi E, Sofia Bernal-Valle, Gustavo A. Gutiérrez-Espeleta

**Affiliations:** 1 Laboratorio de Entomología, Programa de Investigación en Medicina Poblacional, Escuela de Medicina Veterinaria, Universidad Nacional, Heredia, Costa Rica; 2 Escuela de Biología, Universidad de Costa Rica, San Jose, Costa Rica; 3 Departamento de Ecología Humana, Centro de Investigación Y Estudios Avanzados (Cinvestav), Unidad Mérida, Mérida, Yucatan, Mexico; 4 Escuela de Forestales, Instituto Tecnológico de Costa Rica, Cartago, Costa Rica

**Keywords:** Malaria, Molecular diagnosis, Maxent, New World primate, Neotropic

## Abstract

**Background:**

In South and Central America, *Plasmodium malariae/Plasmodium brasilianum, Plasmodium vivax, Plasmodium simium*, and *Plasmodium falciparum* has been reported in New World primates (NWP). Specifically in Costa Rica, the presence of monkeys positive to *P. malariae/P brasilianum* has been identified in both captivity and in the wild. The aim of the present study was to determine the presence of *P. brasilianum*, *P. falciparum*, and *P. vivax*, and the potential distribution of these parasites-infecting NWP from Costa Rica.

**Methods:**

The locations with PCR (Polymerase Chain Reaction) positive results and bioclimatic predictors were used to construct ecological niche models based on a modelling environment that uses the Maxent algorithm, named kuenm, capable to manage diverse settings to better estimate the potential distributions and uncertainty indices of the potential distribution.

**Results:**

PCR analysis for the *Plasmodium* presence was conducted in 384 samples of four primates (Howler monkey [n = 130], White-face monkey [n = 132], Squirrel monkey [n = 50], and red spider monkey [n = 72]), from across Costa Rica. Three *Plasmodium* species were detected in all primate species (*P. falciparum*, *P. malariae*/*P. brasilianum*, and *P. vivax*). Overall, the infection prevalence was 8.9%, but each *Plasmodium* species ranged 2.1–3.4%. The niche model approach showed that the Pacific and the Atlantic coastal regions of Costa Rica presented suitable climatic conditions for parasite infections. However, the central pacific coast has a more trustable prediction for malaria in primates.

**Conclusions:**

The results indicate that the regions with higher suitability for *Plasmodium* transmission in NWP coincide with regions where most human cases have been reported. These regions were also previously identified as areas with high suitability for vector species, suggesting that enzootic and epizootic cycles occur.

**Supplementary Information:**

The online version contains supplementary material available at 10.1186/s12936-021-04036-y.

## Background

Malaria is a vector-borne disease caused by protozoa of the genus *Plasmodium*, mostly transmitted by bites of *Anopheles* mosquitoes. The genus *Plasmodium* includes more than 250 different species that infect mammals, reptiles, birds, and amphibians [1]. Four species are recognized as causing malaria in humans: *Plasmodium falciparum, Plasmodium vivax, Plasmodium malariae* and *Plasmodium ovale.* More recently, *Plasmodium knowlesi* and *Plasmodium cynomolgi* has been implicated as a cause of human malaria [2, 3]. Non-human primates are classified as important hosts of this protozoan, with at least 35 parasite species recognized as simian malaria [4, 5]. This parasite is mostly transmitted by bites of *Anopheles* mosquitoes. There are more than 400 different species of *Anopheles* mosquitoes, and approximately around 30 of these determined as malaria vectors of major importance [6, 7]. In Costa Rica at least 18 species of *Anopheles* were detected, being *Anopheles albimanus* the main vector recognized [8].

*Plasmodium brasilianum* and *P. simium* have been reported in New World primates (NWP) of the Cebidae family and are difficult to distinguish from the human *P. malariae* and *P. vivax,* respectively [9]. *Plasmodium brasilianum* was identified in at least 35 species of NWP in Central and South America [9–11]. *Plasmodium brasilianum* resembles *P. malariae* morphologically, and are nearly genetically identical, differing in mutations expected within a species. Naturally acquired infection with *P. brasilianum* has been found in humans [12]. In Costa Rica Calvo et al. [13] reported the presence of *P. malariae/P. brasilianum* in human samples, the sequences showed a 99% identity with sequences of *Plasmodium* species isolated from NWP in French Guiana. In Costa Rica, the analysis of genetic diversity of the monkey samples positive to malaria revealed that the *P. malariae/P. brasilianum* parasite found in one howler monkey was identical to that recently found in humans residing Costa Rica [11]. *Plasmodium simium* has been determined principally in the Atlantic Forest in southern Brazil, considered specific of NWP, and related, but distinct from *P. vivax*, as established recently [14]. Brasil and collaborators [15] suggest that *P. vivax* outbreaks reported in the Atlantic Forest of Brazil (2015, 2016) were infections caused by *P. simium* transmitted from monkeys to humans by mosquito bites. *Plasmodium falciparum* has been detected in NWP (Aotidae) from Brazil [16, 17], and it is assumed that NWP are susceptible to this malaria parasite [18]. In Costa Rica, *Plasmodium* has been determined both in captivity and in wild NWP. In 2006, the first report of six howler monkeys (*Alouatta palliata*) positive to *P. brasilianum* [10] was published. In 2017 five captive individuals were found positive to *P*. *malariae/P*. *brasilianum* [11].

Worldwide maps of malaria distribution show that Costa Rica is one of the countries with suitable conditions for stable malaria transmission [19]. However, those maps were generated at a coarse scale, so the hotspots in Costa Rica cannot be identified. Recent analysis points out that the Atlantic region of Costa Rica is one of the hot spots of human transmission [20]. Although human cases show a declining trend, there is a concern that enzootic cycles of *Plasmodium* elsewhere in Costa Rica could represent a spillover risk. New World Primates represent interesting populations for study, because of its distribution in areas of ideal weather and altitude in the country for the presence of vector-borne diseases, as is the case for malaria. The constant use of human altered environments by these monkeys as well as its interaction with people [21], may favour the transmission of *Plasmodium* between populations of monkeys or humans by way of mosquito bites. This investigation proposes to determine the presence and potential distribution of climatic suitability for the transmission of *Plasmodium* (*P. malariae/brasilianum, P. vivax and P. falciparum*,) in the four species of natural inhabitants of NWP of Costa Rica: howler (*Alouatta palliata*), white-faced (*Cebus imitator*), squirrel (*Saimiri oerstedii*) and spider (*Ateles geoffroyi*) monkeys. It has been highlighted that infection prevalence and transmission of blood parasites such as avian malaria are affected by environmental conditions and that this information is crucial to develop better risk maps [22]. It is important to emphasize that primate malaria has the potential to damper the efforts to eliminate human malaria.

## Methods

### Study area, sampling size and sampling

The NWP were all collected from 2000 to 2012 in forest areas, and samples from captive animals were gathered from zoos and rescue centres of Costa Rica. The geographic coordinates of each location were obtained using a GPS unit. The animals were captured in situ by chemical immobilization with darts (Type P, 1 mL, Pneu Dart Inc.), and compressed gas rifle (X-Caliber Gauged CO2, Pneu Dart Inc.) for individuals over long distances, or blowgun for individuals located at close range. Anesthetics used were Zoletil 50 (3.3–11 mg/kg) or ketamine (5–20 mg/kg), in combination with Xylazine (0.5–2 mg/kg) [23, 24]. As soon as the animal was anesthetized, a blood sample (2–4 mL) from the femoral, saphene or cephalic vein was taken. The samples were collected in tubes with EDTA and maintained at 4 °C until arrival to the laboratory, where samples were kept at −20 °C until laboratory analysis. The NWP were monitored until awakening from narcosis, and safely released into their habitat.

### Molecular surveys

Total DNA was extracted from all blood samples using the DNeasy Blood & Tissue Kit (Qiagen, Hilden, Germany), according to the manufacturer’s instructions and using 100 ul of blood. Polymerase Chain Reaction (PCR) was carried out as previously described [25], where a sequence of the small ribosomal DNA subunit of *Plasmodium* spp. was amplified. The PCR products were visualized in 2% agarose gel electrophoresis, stained with Gel Red (Biotium Inc., Hayward, California, USA). As positive control *P. malariae*-DNA, *P. vivax*-DNA, and *P. falciparum*-DNA were used, which were donated by the Primatology Institute of the University of Washington, Seattle, USA, and Malaria Reference Center, INCIENSA, Tres Ríos, Costa Rica. Free nuclease water was used as a negative control. Samples with bands of 269 bp (*P. malariae*), 499 bp (*P. vivax*), and 395 bp (*P. falciparum*), were considered positive. Some positive samples were subsequently re-amplified, and the amplicons with adequate concentrations were sequenced with the same diagnostic primers to confirm the PCR results. Sequences derived from this study were aligned together with *Plasmodium* sequences from Genbank then trimmed to a common length of 269 bp, 499 bp, or 395 bp.

### Data assembly and assumptions

Because of the small number of positive samples, all the infection records covering the three *Plasmodium* lineages were lumped together to generate a single supraspecific *Plasmodium* niche model as a valid taxonomic unit to model the ecological niche [26]. This was based on two criteria: (1) the three groups of *Plasmodium* lineages showed no specificity with the four NWP species (see “Results”); and (2) these lineages form a phylogenetically close species group of primates [27]. As a general assumption for the lumping approach, the niche conservatism hypothesis (i.e., niche traits are often conserved among closely related species; [28], were followed which has considerable evidence of lack of ecological niche differentiation at time scales comparable to close/sister species, and states that when niches show high levels of conservatism, close relatives are likely to respond similarly to environmental gradients. This is true not only for free-ranging species, but also for parasites (e. g., [29]). This approach has been assessed under different taxonomic groups that explored the contribution of using supraspecific modelling units in niche construction showing that those modelling units improved the predictive ability of the ecological niche models. Specifically, by adding the occurrences of closer taxa reduced the omission rates considerably [30].

To construct the *Plasmodium* niche model of NWP of Costa Rica, the Maxent's machine learning algorithm [31] was used, the most important algorithm for modelling species' ecological niches and potential distributions. Maxent has been extensively evaluated between other 15 algorithms, and has been highlighted because its high performance, especially with small (less than 20 records) data sets [32, 33]. Due to Maxent uses a generative approach, rather than discriminative, it can perform with a low amount of training data [31]. The state of art modelling package, Kuenm that uses R as a programming environment, and Maxent as a niche model algorithm [34] was used. This package allows robust model calibration processes, facilitating the creation of final models based on model significance, performance, and simplicity. This package allows the user to program different calibration settings, for instance, the use of various sets of bioclimatic predictors, various regularization parameters, which affect the model complexity [35]. By doing this, Kuenm produces thousands of models that can be compared and evaluated.

### Niche model calibration

A total of 21 occurrences of primate individuals that tested positive for *Plasmodium* sp., were included in a primary dataset. Duplicated occurrences were eliminated (e.g., when more than one individual was positive from the same location). Afterwards, the aim was to reduce the effects of spatial autocorrelation by thinning the occurrence of records using a distance filter of 5 km between records with the spThin R package [36]. The final database included 18 occurrence records. Occurrence records were split randomly into two subsets using the “random k-fold” method: 50% of occurrences for model calibration and 50% of occurrences for final evaluation [37]. The latter method partitioned occurrence localities randomly into a user-specified number of (k) bins as described in detail in Muscarella et al. [38].

The accessible area “***M***” is an essential component in the biotic, abiotic, and movement (*BAM*) model [39], that defines the main parameters in constructing the species ecological niche model [40]. To shape this “M” area, a 50 km radius buffer was created around each occurrence point to extend the entire calibration region's limits. Each occurrence record was subsequently overlaid on the ecoregion shapefile to assess the concordance between the species occurrence and a particular ecoregion [41], as described in a previous study [42].

Fifteen variables were included in the modelling framework to construct the ENM of *Plasmodium* spp in Costa Rica. For this, WorldClim 2 was used because WorldClim data performed substantially better than other available climatic data in various modelling purposes (http://www.worldclim.org; [43]). From the whole list of available variables, four variables were excluded from the bioclimatic variables (Bio 8, Bio 9, Bio 18, and Bio 19) owing to their known spatial artifacts, following the protocol implemented in previous similar studies [44]. In order to know whether variable combinations are important to the geographic distribution of niche suitability of infected monkeys, four sets of bioclimatic variables were used that reflect different ecological attributes of the bioclimatic predictors: (a) set1: 15 variables from WorldClim2; (b) set2: nine variables from WorldClim2, a subset of variables with biological relevance defining the availability of thermal energy and water (e.g. the minimum, maximum and mean values of temperature and precipitation); (c) set3: a set of variables selected from a jackknife process in MaxEnt, and correlation analyses to select distinct sets of variables that contributed most to models (> 90%), eliminating one variable per pair with a correlation of (*r*_Pearson_ < 0.8) [37, 45], and d) set4: a set of variables selected after assessing the variance inflation factor (VIF), which is a measure of levels of multicollinearity between pairs of variables. Values of VIF > 10 denote a potentially problematic correlation with of covariates, indicating that these covariates should be carefully evaluated in model development [46]. All bioclimatic variables used are at the spatial resolution of 30 arc seconds (≈1km2 per pixel) (Table 1).

**Table 1:** Set of Bioclim variables used for the construction of the ecological niche modelling for *Plasmodium* spp

Bioclimatic variables	Code	Set 1	Set 2	Set 3	Set 4
Annual mean temperature	Bio01	X	X		
Mean diurnal range	Bio02	X			
Isothermality	Bio03	X		X	
Temperature seasonality	Bio04	X	X		X
Max temperature of warmest month	Bio05	X	X		
Min temperature of coldest month	Bio06	X	X		
Temperature annual range	Bio07	X	X		
Mean temperature of warmest quarter	Bio10	X		X	
Mean temperature of coldest quarter	Bio11	X		X	
Annual precipitation	Bio12	X	X		X
Precipitation of Wettest month	Bio13	X	X		X
Precipitation of driest month	Bio14	X	X	X	X
Precipitation seasonality	Bio15	X	X		
Precipitation of wettest quarter	Bio16	X			
Precipitation of driest quarter	Bio17	X			X

ENM was constructed using the maximum entropy algorithm implemented in MaxEnt version 3.4.1 via the *kuenm* R package [34]. In the Kuenm package, it is possible to configure the modelling procedure by including different parameterizations to leave the program to construct several models that can be assessed by various criteria (see below). To do this, candidate models were created by combining the four sets of environmental variables, 17 values of regularization multipliers (1, 2, 3, 4, 5, 6, 7, 8, 9, 10), and all 29 possible combinations of 5 feature classes (linear = l, quadratic = q, product = p, threshold = t, and hinge = h) [34]. The best candidate model was selected based on three criteria: (1) significance, (2) performance, and (3) the Akaike information criteria (AIC): AICc, delta AICc, and AICc weights. The criteria used to assess the model performance and select the best candidate models included first their statistical significance: partial Receiver Operating Characteristic (partial ROC) and omission rates 5% for predictive ability; and second by performance based on Akaike Information Criterion corrected (AICc) for small sample sizes [47]. Partial ROC assesses a ratio from the number of evaluation occurrences predicted correctly, and the proportion of area predicted suitable—a ratio with values ≤ 1 reflects predictions indistinguishable from random predictions, while a ratio > 1 suggests predictions better than by random [48].

The omission rate is a threshold that considers an estimate of the likely amount of error among occurrence data and thus removes 5% of occurrences with the lowest suitability values (*E* = 5%) [48]. The AICc provides explicit criteria for selecting models of appropriate complexity. This metric is assessed by standardizing raw scores for each ENM so that all scores within the geographic space sum to 1, and then calculating the likelihood of the data given each ENM by taking the product of the suitability scores for each grid cell containing a presence. Both training and test localities are used in calculating likelihoods. The number of parameters is measured simply by counting all parameters with a nonzero weight in the lambda file produced by Maxent, a small text file containing model details that Maxent produces as part of the modelling process [35]. Models were selected with delta AICc ≤ 2 from those that were statistically significant, and had omission rates below 5% [34]. The criteria followed was from a previous study [34] to select the final model, evaluate the model, and assess extrapolation risk. The final models of *Plasmodium* spp were created using ten replicates by bootstrap, with logistic outputs, and these models were transferred from the accessible area “***M***” to the projection area “***G***.”

To identify extrapolation risk in the model transfers, a mobility-oriented parity (MOP) analysis was performed comparing the environmental breadth of predictors within “***M***” (10% reference points sampled) with that in the projection area using the MOP function [34] available in the *kuenm* R package. The risk of extrapolation analysis defines the areas with strict extrapolation (i.e., places where environmental conditions are non-analogous to those in regions across which the models were calibrated) to avoid the risk of over-prediction in non-analogous environments.

## Results

**Fig. 1:**
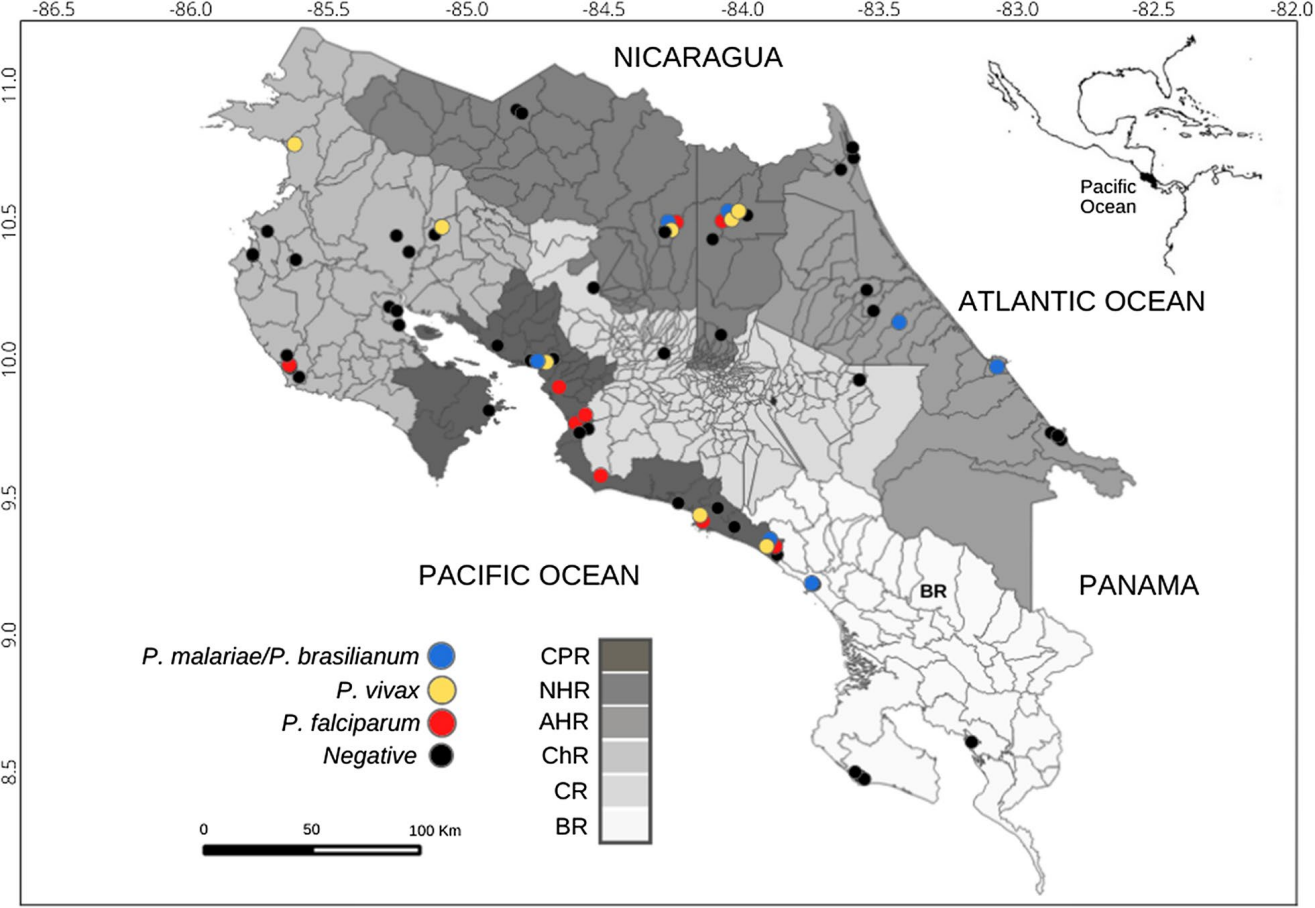
Map of *Plasmodium malariae/Plasmodium brasilanum*, *Plasmodium vivax*, and *Plasmodium falciparum* in positive New World primates from Costa Rica, 2000–2012. Regions: CPR—Central Pacific Region, NHR—Northern Huetar region, AHR—Atlantic Huetar region, ChR—Chorotega region, CR—Central Region, BR—Brunca Region

**Fig. 2:**
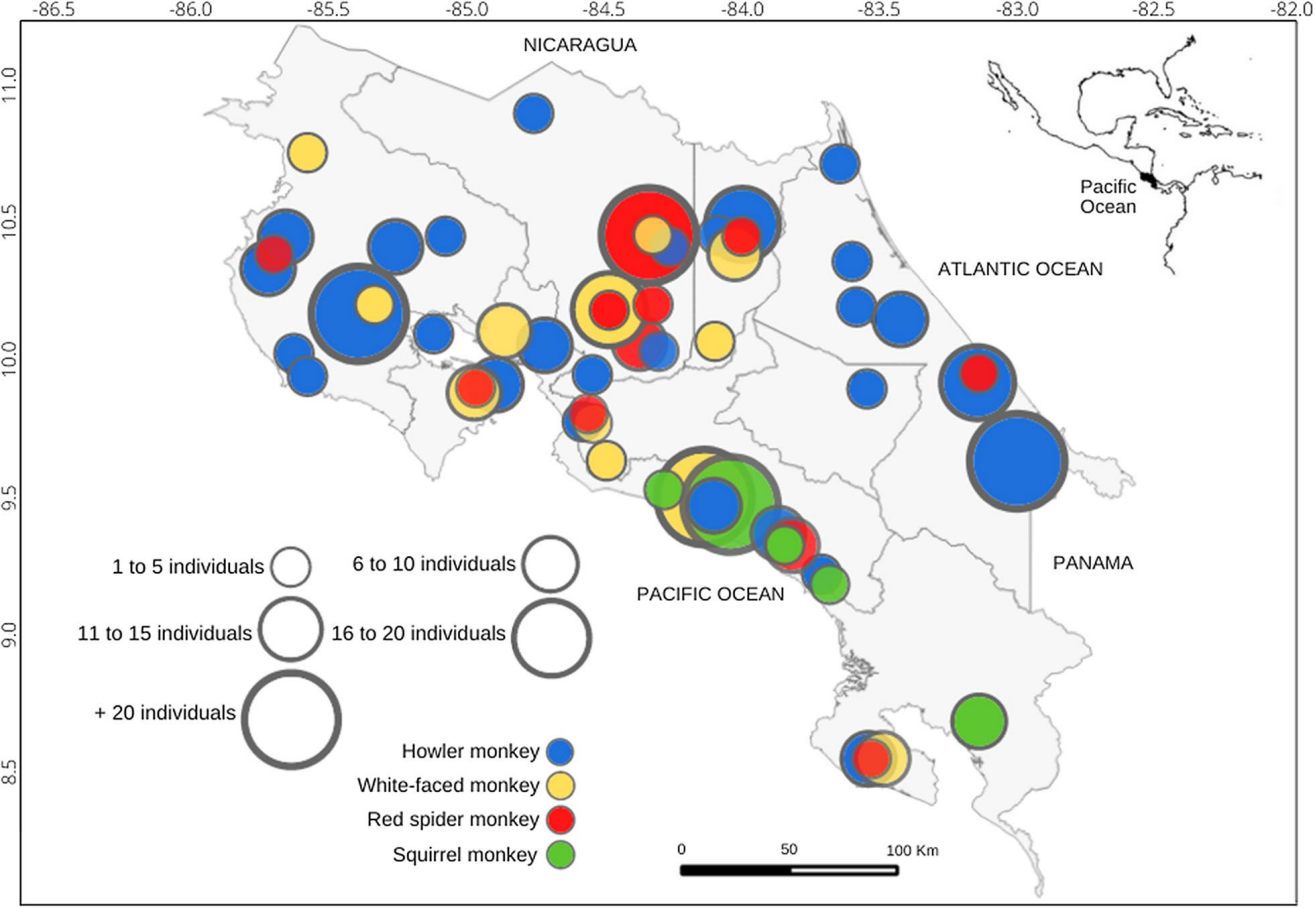
Map of sampling sites of New World primates from Costa Rica, 2000–2012

Throughout the study area, 384 individuals from the four species of NWP were sampled during surveys: 130 howler monkeys (*Alouatta palliata*), 132 white-faced monkeys (*Cebus imitator*), 50 squirrel monkeys (*Saimiri oerstedii*), and 72 red spider monkeys (*Ateles geoffroyi*) (Fig. 1). A total of 342 (89%) and 42 (11%) individuals were captured in free-range and captive areas, respectively. Of the 384 NWP studied 8.6% had evidence of *Plasmodium* presence*,* seven captive and 26 free-ranging individuals. Of the positive individuals, 3.4% were positive to *P. falciparum*, 3.1% to *P. malariae/P. brasilianum,* and 2.1% to *P. vivax*. The PCR products showed nucleotide sequence similarity to *P. malariae*, *P. vivax*, and *P. falciparum*, analysis to corroborate positive results (data not shown). The four species of NWP were found positive. *Plasmodium* positivity by monkey species was the following: howler (7.7%), white-faced (7.6%), squirrel (10%), and red spider (11.1%) (Table 2, Fig. 2). An increase in the number of positives was determined over time. Of the positive individuals seven (21%) were captive and 26 (79%) free ranging. The seven positive captive individuals were red spider monkeys, one (*P. falciparum*) in the Central Pacific region of Costa Rica in 2010, and six (three *P. falciparum*, two *P. malariae*, and one *P. vivax*) living in the Northern regions in 2005 (Table 2).

**Table 2:** New World primates and locations found *Plasmodium* positive, and species detected in Costa Rica, 2000–2012

Species	Year	Sample number		*P. falciparum*	*P. malariae/P. brasilianum*	*P. vivax*	Total +	Positive region
		Wild	Captivity					
	2000	3	0	0	0	0	0	
Howler monkey	2001	29	0	0	0	0	0	
	2002	43	0	0	0	0	0	
	2007	1	0	1	0	0	1	Northern Huetar region
	2008	14	0	0	4	0	4	Atlantic Huetar region
	2009	9	0	0	0	0	0	
	2010	17	0	2	0	2	4	Chorotega (1), Northern Huetar (1), Central Pacific (2) regions
	2011	15	0	0	0	1	1	Chorotega region
	2012	2	0	0	0	0	0	
	Total	130		3	4	3	10	
White-face monkey	2001	9	0	0	0	0	0	
	2002	6	0	0	0	0	0	
	2003	19	0	0	0	0	0	
	2004	23	0	1	0	0	1	Central Pacific region
	2005	8	0	0	1	0	1	Northern Huetar region
	2006	10	0	0	3	0	3	Brunca (1); Central Pacific (2) regions
	2007	4	0	0	0	0	0	
	2010	6	4	1	0	0	1	Central Pacific region
	2011	24	1	2	0	2	4	Chorotega (1) Central Pacific (3) regions
	2012	10	8	0	0	0	0	
	Total	132		4	4	2	10	
Squirrel monkey	2002	7	0	0	0	1	1	Central Pacific region
	2003	14	0	0	0	0	0	
	2004	9	0	0	0	0	0	
	2006	9	0	1	2	0	3	Central Pacific region
	2007	3	0	0	0	0	0	
	2010	4	0	0	0	0	0	
	2011	4	0	1	0	0	1	Central Pacific region
	Total	50		2	2	1	5	
Red spider monkey	2001	4	0	0	0	0	0	
	2004	2	3	0	0	0	0	
	2005	20	8	3^a^	2^a^	2^a^	7	Northern Huetar region
	2006	17	4	0	0	0	0	
	2007	0	6	0	0	0	0	
	2010	0	1	1^a^	0	0	1	Central Pacific region
	2011	0	6	0	0	0	0	
	2012	0	1	0	0	0	0	
	Total	72		4	2	2	8	
Final total		384		13 (3.4%)	12 (3.1%)	8 (2.1%)	33 (8.6%)	

^a^Captivity positive

**Fig. 3:**
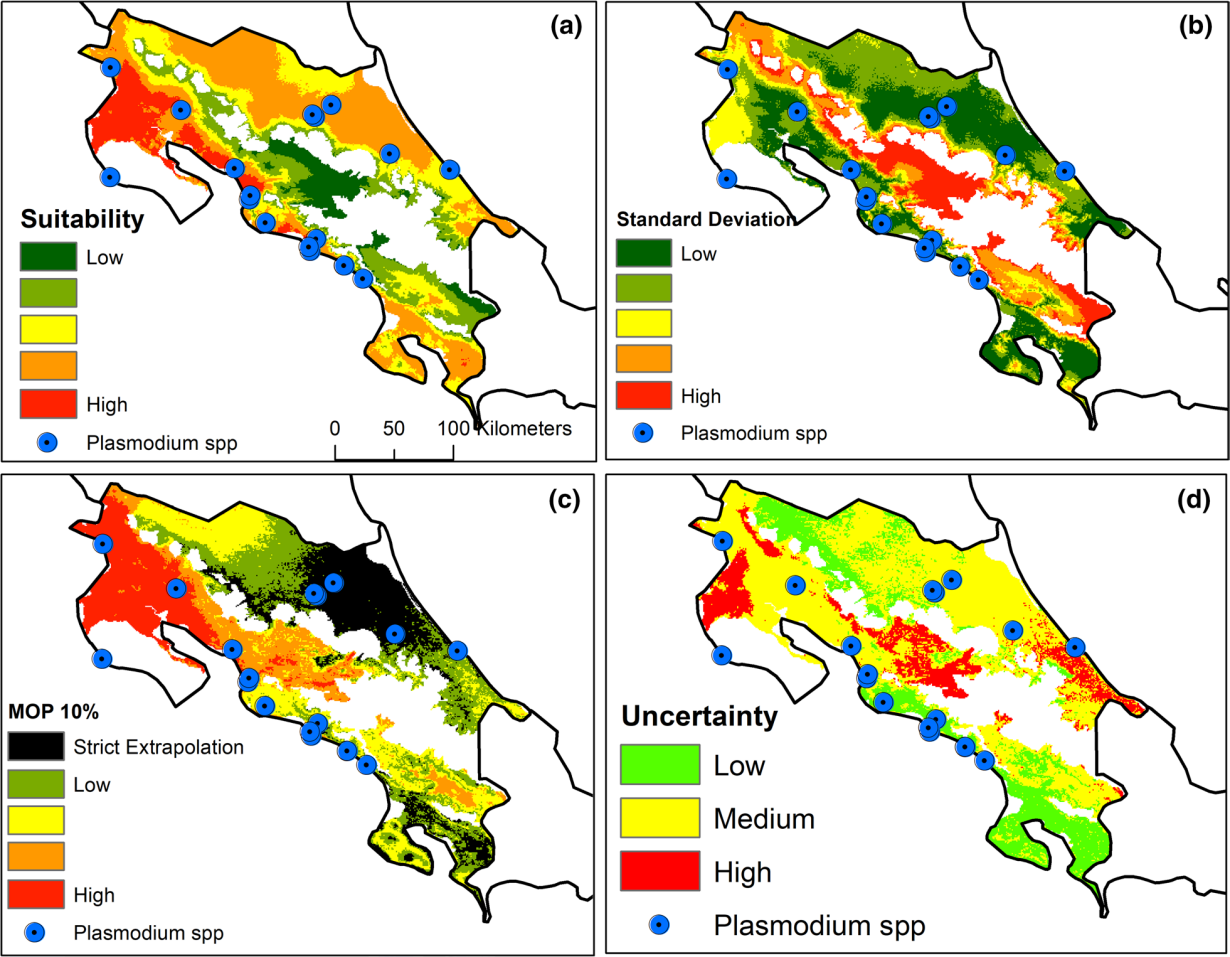
Map of the 1st best model (see Table 1) and uncertainty for *Plasmodium* sp. In Costa Rica. **a** Ecological niche model of Plasmodium sp. In Costa Rica; **b** Standard deviation map; **c** Mobility-oriented parity (MOP) extrapolation risk analysis for the ecological niche model (see “Methods”); **d** Uncertainty map of *Plasmodium* sp. prediction

The modelling framework used produced 1240 candidate models, of which 747 were statistically significant, and only six passed the three selection criteria (Table 3; Additional file 1: Fig. S1). The best models used set 2 of bioclimatic predictors (i.e., the subset of variables with biological relevance). From these six models (Additional file 2: Fig. S2), model 2 (Fig. 3) was chosen to depict the potential distribution of *Plasmodium* species in Costa Rica. The Pacific and the Atlantic coastal regions of Costa Rica presented suitable conditions for parasite infections (Fig. 3a); however, the Atlantic coast showed a higher variation between the chosen model's replicate outputs (Fig. 3b). This can result from a non-analogous climatic envelope to the rest of the country, as shown in the MOP analysis (Fig. 3c). The uncertainty map shows that the central pacific coast has a more trustable prediction for malaria in primates (Fig. 3d).

**Table 3:** Model performance under different parametrizing settings (i.e. regularization multiplier [RM] and features classes [FC]

Best Models	RM	FC*	p.ROC	O.rate 5%	AICCc	∆AICCc	#
1th	4	p	1.36	0.00	287.50	0.00	1
2th	4	pt	1.36	0.00	287.59	0.00	1
3th	5	lp	1.37	0.00	287.92	0.41	1
4th	5	lpt	1.37	0.00	287.92	0.41	1
5th	5	p	1.37	0.00	287.92	0.41	1
6th	5	pt	1.37	0.00	287.92	0.41	1

Model evaluation used Mean AUC ratio (AUC.r), partial ROC (p.ROC), omission rate 5% (O. rate5%), Akaike Information Criterion corrected (AICCc), Delta Akaike Information Criterion corrected (∆AICCc), Akaike Information Criterion corrected weight (AICCc.W) and number parameters (#) for modelling ecological niche for *Plasmodium sp*. * l = Linear; q = Quadratic; t = Threshold; h = Hinge; p = Product

## Discussion

The model of potential distribution of *Plasmodium* transmission in NWP of Costa Rica, indicates a higher niche suitability for the transmission in the North and Central Pacific and moderate suitability in Atlantic regions, near to where *P. malariae*/*P. brasilianum* was reported in primates living in captivity by Fuentes-Ramírez and collaborators [11] (Central Pacific, and Atlantic regions). Yet, since the Atlantic region showed high to moderate model uncertainty in the analysis, the transmission prediction in free-ranging primates in this area should be taken with caution. While the model used showed a relative week prediction for primate prediction for the Atlantic region, Chinchilla and collaborators [10], have reported the presence of *P. brasilianum* in howler monkeys in the Atlantic region, and with areas where there have been most reports of human malaria cases in Costa Rica [49], and areas of new autochthonous outbreaks in the country [50]. On the Pacific coast, the model of distribution indicates a high probability of occurrence in regions where the presence of evolutive stages of *Plasmodium* has been previously reported [49]. Therefore, the presence of *Plasmodium* in NWP in Costa Rica coincides with areas known as malarious for Costa Rica. Areas of incidence of malaria cases in humans will be considered given the agent's ability to infect both human and non-human primates [51]. It is possible that a high incidence of the disease in humans may reflect a high presence of positive NWP in close contact with humans, which can facilitate transmission by the vector.

The presence over time showed an increase in positive individuals, which is contrary that reported in humans, where the number of cases were decreasing during the period of this investigation [8]. Costa Rica has been a country of historically low malaria incidence. The incidence of human malaria has declined in the country since 1999. However, in recent years a greater number of imported cases have been reported, and in 2017 after five years of no reported cases native to Costa Rica [50], the Costa Rican Ministry of Health declared a sanitary alert for areas 500 m above sea level.

*Plasmodium* is a common agent in non-human primates, and in diverse environmental areas, that can benefit from anthropogenic changes [9]. The study of potential distribution allows the acquisition of knowledge about the factors associated with the temporal and spatial variation of agents such as *Plasmodium*. The great variety of microclimates and life zones of Costa Rica maintain one of the highest levels of species diversity on the planet. The country has become known for its conservation efforts. However, the forests of Costa Rica face risks from illegal logging, forest conversion for agriculture (such as pineapple and palm oil production), and cattle pasture in unprotected areas, as well as tourism and real estate development disrupting the connectivity between forest patches. Land use and climate change favour the presence of potential vectors and affect the composition of wildlife communities, which influence human and animal exposure to the parasite as well as its prevalence both at a spatial and temporal level [9]. A definite pattern with the diversity and proportion of *Plasmodium* species throughout the years was not observed, *P. falciparum* was the species most often found, followed by *P. malariae/P.brasilianum*, and *P. vivax*. When compared to the cases reported in humans of Costa Rica, most human cases reported are *P. vivax*, followed by *P. falciparum*, and specifically *P. malariae/P. brasilianum* cases are very rare [49], and were reported for the first time in 2015 after almost 40 years [13]. It is not possible to define a similar pattern of incidence of *Plasmodium* infections in NWP and humans, although a greater presence of positive individuals in areas of historically high incidence of human cases [20] was determined.

In agreement with the proposed possibility that non-human primates may be natural reservoirs for human malarias [52, 53], *P. falciparum* and *P. vivax* have not been reported in NWP of Costa Rica. The presence of *P. falciparum* in NWP has already been reported in Brazil [16, 17], and a possible frequent transmission of the human agent to monkeys was determined. *Plasmodium vivax* was the most often detected species in human cases of Costa Rica. In 2007, 99% of reported malaria cases were *P. vivax*, and the remaining 1% was *P. falciparum* [49]. *Plasmodium simium,* a sister species of *P. vivax*, has been reported to date only in NWP of Brazil*,* and was recently determined as a monkey specific species, distinct from *P. vivax*, by a differential diagnosis developed for both species [14] (de Alvarenga et al. 2018). Oliveira et al. [54] propose a process of speciation is occurring in *P. simium* which has adapted to sylvatic reservoirs, but can infect humans due to the presence of vectors such as *Anopheles cruzii* with the ability to feed on both monkeys and humans. Outbreaks of malaria diagnosed in 2015 and 2016 in Costa Rica were reported as *P. vivax*, it is important to investigate in future studies if native cases reported in the Atlantic region, were infections caused by *P. vivax* or by *P. simium,* transmitted from monkeys to humans by mosquito bites [15]. In Costa Rica, the analysis of genetic diversity of the monkey samples positive to malaria revealed that the *P*. *malariae* parasite found in one howler monkey was identical to that recently found in humans residing Costa Rica [11]. For this reason and considering the proven experimental replication of this *Plasmodium* in NWP (Aotidae and Cebidae) [55–57] it is relevant to determine the possibility that these species of monkeys can maintain the parasite cycle in sylvatic environments, considering the great diversity of *Anopheles* present in Costa Rica [8], which may act as malaria vectors for NWP.

It is expected that the vectors between monkeys and humans differ in their distribution by the habitats used [16]. Although *An. albimanus* is the main vector associated with the transmission of malaria in humans in Costa Rica [8], the presence of at least 18 *Anopheles* species has been reported [58]. Including *An. neivai* (*Kerteszia* subgenus) associated with the transmission of malaria in NWP and humans in Brazil, since its ability to be found both in the forest canopy and at ground level [59]. All positive individuals are in environments where the constant interaction between humans and NWM is commutated. Only one location with positive individuals was not in a fragmented environment, and all areas with positive cases are close to villages. This may be important, because although vectors between humans and wild animals are normally not shared, it has been established that proximity between urban and conserved areas have favoured humans and monkeys sharing malaria vectors [60, 61].

## Conclusions

Here in, a first geographical approach was developed to understand the spatial patterns of malaria transmission in NWP and how this can be related with human malaria in Costa Rica. The modelling framework used allowed for the identification of areas where spillover is possible providing the coincidence between areas with climatic suitability for the primate parasite transmission with the presence of human populations. Modelling parasite-infected or diseased individuals is a challenging task since the natural infection prevalence is often low. In the presented case, several records were available of monkeys, but only 8% of infection prevalence, so there was a reduced dataset for model calibration. Although a straightforward framework was used to reduce the effect of sampling size on model quality, it was considered that for some regions the predictions must be taken with caution (i.e., in the Atlantic region) based on a limited representation. Since maps were produced for model uncertainty and the extrapolation risk, areas were identified where the model used was more or less robust. The authors encourage more research to document the spatial locations where primate infections with *Plasmodium* occur in order to a better to enhance the understanding of the relationship between the geographical and environmental correlates with malaria transmission, that can help plan more effective control strategies, using the natural environment cycles as disease sentinels.

Due to the great diversity of *Anopheles* in Latin America [7], it is a necessity to carry out studies that allow for comparing the species and abundance of *Anopheles* in places where NWPs are distributed. As well integrate the taxonomic determination of mosquitoes and infectivity for *Plasmodium* into research to establish which of these species are acting as malaria vectors for NWP, and to confirm the presence of *P. simium* in the country. Finally, it is recommended that an exhaustive comparison be made between free-living individuals and individuals kept in captivity to determine if captivity favours the presence of positive individuals.

## Supplementary Information

**Additional file 1: Fig. S1.** Omission rates and AICc values for all, non-significant, and selected “best” candidate models for the *Plasmodium* sp.

**Additional file 2: Fig. S2.** Maps of the best models according to the performance evaluation. Maps order are the same as Table 1, (a) 1st, (b) 2nd, (c) 3rd, (d) 4th, (e) 5th, and (f) 6th.

## Data Availability

The datasets used and/or analyzed during the current study are available from the corresponding author on reasonable request.

## Abbreviations

NWP: New World primates
PCR: Polymerase chain reaction
mg: Milligram
kg: Kilogram
mL: Millilitre
°C: Degree centigrade
bp: Base pairs
VIF: Variance inflation factor
ROC: Receiver operating characteristic
AICc: Akaike information criterion corrected
