# Supplementary figures and images for: Presence and potential distribution of malaria-infected New World primates of Costa Rica
Source: Malar J. 2022 Jan 8;21:17. doi: 10.1186/s12936-021-04036-y (PMC8742953; doi:10.1186/s12936-021-04036-y)

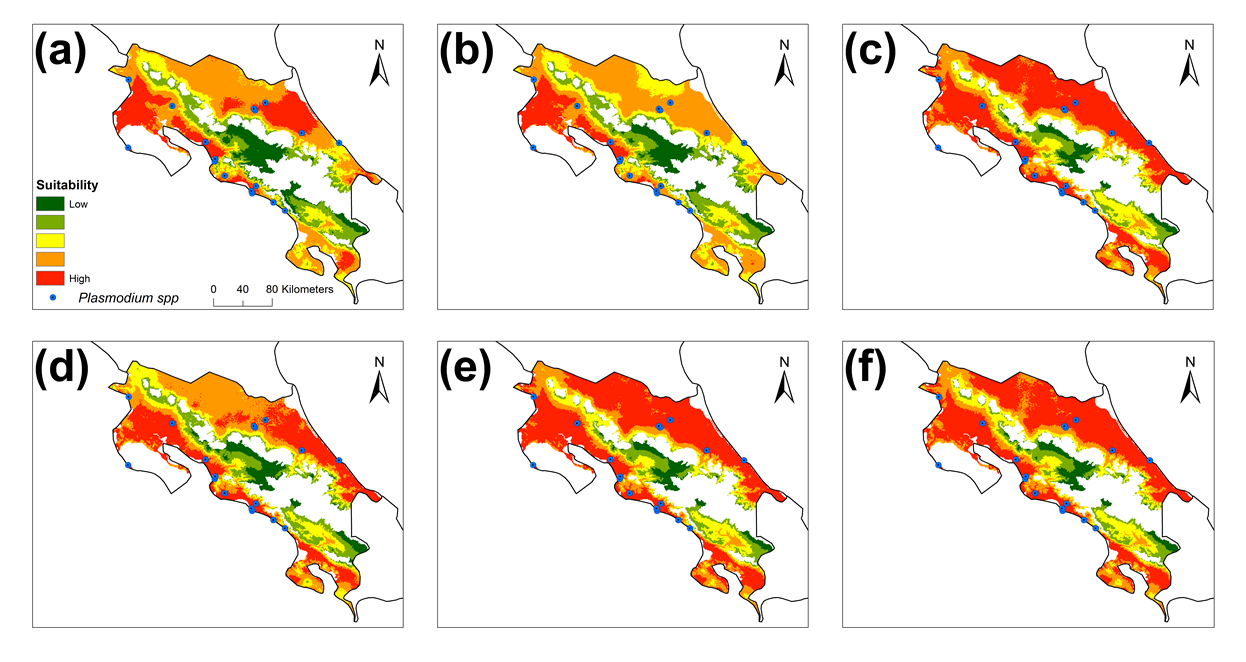

Supplement: Supplementary file 2 — Additional file 2: Fig. S2. Maps of the best models according to the performance evaluation. Maps order are the same as Table 1, (a) 1st, (b) 2nd, (c) 3rd, (d) 4th, (e) 5th, and (f) 6th. [file 12936_2021_4036_MOESM2_ESM.tif]
